# Technological and Probiotic Traits of the Lactobacilli Isolated From Vaginal Tract of the Healthy Women for Probiotic Use

**DOI:** 10.15171/ijb.1432

**Published:** 2016-09

**Authors:** Hamida Bouridane, Mohamed Sifour, Tayeb Idoui, Lejeune Annick, Philip Thonard

**Affiliations:** ^1^Laboratoryof Biotechnology, Environment and Health, University Mohammed Seddik Benyahia, Jijel, Algeria; ^2^Department of Applied Microbiology and Food Sciences, Faculty of Sciences, University Mohammed Seddik Benyahia, Jijel, Algeria; ^3^Bio-Industries Unit CWBI, Gembloux Agro. Bio-Tech, University of Liege, Passage Deportees, Gembloux, Belgium; ^4^Laboratory of Molecular Toxicology, Faculty of Sciences, University Mohammed Seddik Benyahia, Jijel, Algeria

**Keywords:** Adherence, Aggregation, *In vitro*, Lactobacilli, PCR, Vaginal tract

## Abstract

**Background:**

For biotechnological application, selected lactic acid bacteria strains belonging to the genera *Lactobacillus (Lb)* are proposed as an alternative to the antibiotics for the prevention and treatment of urogenital tract infections.

**Objectives:**

Isolating and selecting vaginal lactobacilli strains for probiotic use based on their technological and probiotic aptitudes.

**Materials and Methods:**

The vaginal isolates were examined for their essential characteristics as the potential probiotic such as low pH tolerance, bile-salt and simulated human intestinal fluid (SIF) resistance, adhesion to the vaginal epithelial cells (VECs), aggregation and coaggregation, surface hydrophobicity, antimicrobial activity, acid production, antibiotic resistance, and resistance to spermicides. The best strain was identified by PCR.

**Results:**

From 70 lactobacilli isolates and according to the 16 rDNA sequences, isolates B6 and B10 showed the closest homology (99%) to the *Lb. gasseri* and *Lb. plantarum* respectively. They produced hydrogen peroxide (H_2_O_2_), tolerant to acid, bile, simulated human intestinal fluid, present a strong adhesion, highest percentages of aggregation, and antibacterial activity. These strains are resistant to the spermicide and actively acidify the growth medium.

**Conclusions:**

Strains *Lb. plantarum* B10 and *Lb. gasseri* B6 have a strong potential probiotic confirming their value as a tool for prevention against urinary and vaginal infections.

## 1. Background

Urogenital tract infections (UGTI) and sexually transmitted infections are the two major medical problems, affecting millions of women every year (1-3). Although, antibiotic and drug therapy can be effective for the eradication of the urinary tract infection, however several problems have emerged, such as multiresistant bacteria (4-6). For the prevention and treatment of UGTI, probiotic lactobacilli were proposed as an alternative to the antibiotics (7,8). Moreover, vaginal lactobacilli have a number of properties which render them highly suitable for probiotic (5,9). A healthy vaginal women microbiota is dominated by lactobacilli. They play a significant role in the maintenance of ecological balance (10,11). The protective role of lactobacilli is based on several mechanisms; specific adherence to the vaginal epithelium and inhibition of the pathogens adhesion to the vaginal epithelial surface, coaggregatin with some uropathogenic bacteria , competition for nutrients, production of the active metabolites including organic acid, mainly lactic acid that contributes in the maintenance of the low vaginal pH (4-4.5), hydrogen peroxide (H_2_O_2_) is usually generated by lactobacilli present in a healthy vagina, production of bacteriocins (5,10-12), but there are significant differences between strains (14). However, there are only few reports concerning probiotics residence in the female reproductive tract, although the presence of lactobacilli in vagina was observed by Doderlein in 1841 (6).

The aim of this study was to get a better knowledge on the potential probiotic properties of the vaginal lactobacilli of the healthy Jijelian women (Algeria) in order to gain new indications on how to improve their exploitation in new the technological applications in probiotic production.

## 2. Objectives

The main goal of the present work was to isolate lactobacilli strains from vaginal samples and to investigate their probiotic properties.

## 3. Materials and Methods

### 3.1. Vaginal Samples

Vaginal specimens were obtained from 60 women (19-46 years) with healthy vaginal ecosystem of Jijel region. From vaginal fluid, samples were collected with sterile cotton swabs inserted into the vagina, rotated a few turns along the vaginal sidewall, and allowed to absorb for few seconds (15). The swabs were immersed in the sterile normal saline, and used for anaerobic (BD Gaspak^TM^ Anaerobic system, USA) culture preparation by streaking on de Man Rogasa Sharpe (MRS) Agar (CONDA, Pronadisa, Spain) plates (16). The same swab was used to prepare a smear, underwent Gram staining, and was evaluated by using Nugent criteria. The flora was interpreted as normal (score 0-3), as intermediate (score 4-6), and interpreted as consistent with BV (score 7-10) (17).

### 3.2. Isolation and Identification of Microorganisms

The swabs were vortexed for 1 min at maximum speed, the resultants suspensions were diluted 7 times with potassium phosphate buffer (pH 7.4), and 50 μL of each dilution was seeded on MRS agar plate (pH 5.5). The plates were incubated for 48 h at 37ºC under anaerobic condition (16,18). To identify strains, each colony was subjected to colony morphology, Gram coloration, catalase activity test, motility, growth at various temperatures, and homofermentative/heterofermentative test. They were further characterized by the carbohydrate fermentation, then stored at -20ºC in MRS broth supplemented with glycerol (30% v/v) (13,19).

### 3.3. Determination of Hydrogen Peroxide Produced by Lactobacilli

Isolates were cultured into MRS agar medium supplemented with 250 μg.mL^-1^ 3,3´,5,5´-tetramethylbenzidine (Sigma-Aldrich, USA) and 0.01 mg.mL^-1^ horseradish peroxidase (Sigma-Aldrich, USA), incubated in an anaerobic condition at 37ºC for 48 h. The plates were exposed to aerobic environment for 30 min. The color intensity was graded the potential of H_2_O_2_ production(20,21).

### 3.4. Determination of Acid and Bile Salt Tolerance

Fourteen (14) isolates (H_2_O_2_^+^) were cultured for 16-18 h and the cells were collected by centrifugation (30000 ×g, 15 min). The pellet were washed twice and finally suspended in phosphate buffer saline (PBS). Approximately 10^8^ CFU.mL^-1^ of each isolate was inoculated into the acidified PBS pH 2.0 and incubated for up 2 h. The viable cells were counted at 0 h and after 2 h (22). The bile tolerance of each isolate was determined by comparing the count after 8 h of exposure to 0.3% bile salt Oxgall (Sigma-Aldrich, USA), (w/.v) with initial count at 0 h (22).

### 3.5. Survival in Simulated Human Intestinal Fluid

Simulated intestinal fluid (SIF) was prepared with 9 g.L^-1^ NaCl, 10 g.L^-1^ of pancreatin, 10 g.L^-1^ trypsin and 3 g.L^-1^ of bile salts (pH 6.5). The cultures were incubated in this solution for 180 min at 37ºC. The number of viable cells was counted at 0 h, 90 min and after 180 min (23).

### 3.6. Adhesion to Vaginal Epithelial Cells (VECs)

VECs were collected from healthy premenopausal volunteers’ women by sterile cotton swabs, immersed in 0.04 M citric acid -Na2HPO4 buffer pH 4.5 and stored at 4ºC for less than 3 h until use. The VECs were washed using the same buffer, centrifuged at 800×g for 4 min and resuspended to a concentration of 1×106 VEC.mL^-1^ (24). An overnight culture of the lactobacilli was suspended to reach 10^8^ CFU.mL^-1^ in normal saline. Equal volumes of the bacterial suspension and the vaginal cells were mixed and incubated for 1h at 37ºC and the cells with adherent bacteria were collected and washed three times in citric-acid- Na2H2PO4 buffer (800 ×g, 7min). Bacterial adhesion to VECs was assessed by microscopy (×100) after staining with 1% of crystal violet. The number of bacteria attached to 50 consecutive VECs smears was counted and VECs Controls smears were made to confirm that the presence of native bacteria was negligible (15).

### 3.7. Growth Inhibition of Vaginal and Urogenital Pathogens

Indicator strains used were *E. coli* ATCC25922, *Staphylococcus aureus* ATCC25923, *Klebsiella pneumonia* ATCC700603 (UHC, Constantine, Algeria), vaginal strains (*E. coli*, *Staphylococcus aureus*, *Candida albicans*) and urogenital strains (*E. coli*, *Klebsiella*. sp).

Inhibitory activity was determined by well diffusion test with minor modifications (17). The surface of Muller-Hinton agar plate was spread with a standardized suspension of each pathogen microorganisms (107CFU.mL^-1^). Culture supernatants of the lactobacilli isolates were filtered through 0.45 μm cellulose filter (Sartorius, Germany). 25 μL of the cultures and supernatants of lactobacilli were placed into wells in the pathogens inoculated plates and were incubated for 24 h at 37ºC. The diameter of inhibition zones was measured. Control assays of MRS medium with pH 6.5 and pH 4.0 were also performed (5).

### 3.8. Autoaggregation and Coaggregation Assay

Bacterial cells of overnight culture were harvested by centrifugation at 5000 ×g for 15 min, washed twice in PBS pH 6.0 to give viable counts of 10^8^ CFU.mL^-1^. Four ml of cell suspension were mixed by vortexing for 10 s and autoaggregation was determined during 5 h of incubation at room temperature. 0.1 mL of the upper suspension was transferred to another tube containing 3.9 mL PBS and the absorbance was measured at 600 nm (25). The percentage of autoaggregation was calculated by the following expression:

Autoaggregation (%) = [OD_i_ - OD_f_ / OD_i_] ×100

Where OD_i_ is the OD at initial time (t=0 h) of autoaggregation assay, and OD_f_ is the OD at t=1 h, 2 h, 3 h, 4 h and 5 h.

For the co-aggregation experiment, equal volumes (2 mL) of the lactobacilli and indicator strain cultures were mixed together by vortexing for 10 s. 4 mL of each bacterial suspension were used as a control. The absorbance was measured at 600 nm after mixing and 5 h of incubation at room temperature (25). The percentage of coaggregation was calculated using the following equation:

$$\text{\% Coaggregation}=\frac{{\text{[(A}}_{\text{x}}{\text{+A}}_{\text{y}}\text{)/2] - A (x+y)}}{{\text{[A}}_{\text{x}}{\text{+A}}_{\text{y}}\text{]/2}}\times 100$$

x and y represent strains in the control tube and (x+y) the mixture.

### 3.9. Hydrophobic Partition

Bacterial pellets were obtained from overnight cultures, washed, and resuspended in urea magnesium phosphate buffer (pH 6.0). The absorbance of the cell suspension was measured at 450 nm to obtain approximately 1.0. Three mL of the bacterial suspensions were put in contact with 0.6 mL of xylene and vortexed for 2 min. The lower aqueous layer was carefully removed, transferred to the clean tubes, and absorbance was measured as described before (26). The percentage of hydrophobicity was obtained from the following calculation:

% hydrophobicity= [(OD_before_-OD_after_)]/OD_before_ ×100

### 3.10. Acid Production

Lactobacilli strains were inoculated (1%) in MRS broth (pH 6.5) and the amount of acids produced was indirectly determined by measuring the pH of culture supernatants with a pH meter (HANNA, HI 2211/ PH/ORP Meter). The experiments were performed in duplicate and the mean pH±S.D was calculated (9).

### 3.11. Antibiotic Resistance

The antibiotics used were inhibitors of the cell wall synthesis such as Ampicillin (10 μg), Cefotaxime (30 μg), inhibitor of protein synthesis (Gentamicin (10 μg), Erythromycin (15 μg), Chloramphenicol (30 μg), Tetracycline (30 μg) and inhibitor of nucleic acid synthesis (Ofloxacin, (5 μg), Trimethoprim-sulphamethoxazole (25 μg), Ciprofloxacin (5 μg), Nitrofurantoin (200 μg). Antibiotic discs were obtained from (Bioanalyse®). The disk diffusion method was used. After incubation the diameters of the inhibition zone were measured (5,27).

### 3.12. Resistance to Spermicides

Suspensions of 14 isolates were adjusted to 10^8^ CFU.mL^-1^ and then were inoculated by swabbing onto MRS agar. 10 μL of each concentration (0.1%, 0.25%, 1%, and 2.5%) of nonoxynol-9 (N-9) cream was deposited on each disk. After incubation, the strain was considered sensitive if the diameter of the inhibition zone is more than 9 mm (20).

### 3.13. Genetic Identification of the Best Lactobacilli

To identify the most efficient isolates, 16S Ribosomal DNA (16S-rDNA) sequencing was performed. Genomic DNA was extracted with the Wizard® genomic DNA purification kit (Promega, Madison, USA) according to the manufacturer instruction. To amplify 16S-rDNA, polymerase chain reaction (PCR) was used with the following primers 16SP0: 5´-GAAGAGTTTGATCCTGGCTCAG- 3´ and 16SP65´-CTACG-GCTACCTTGTTACGA-3´. Fragment of about 1500 bp in size was excised from a 1% (w.v) agarose gel staining and purified with Microcon YM-100 kit (Bedford, MA, USA). The BigDye Terminator sequence was performed using the Vector NTI (Version 8) software package (BD Biosciences, San Jose, USA). The sequences were compared with sequences deposited in the Gen Bank database of the National Center for Biotechnology Information using the BLAST program. The phylogenetic tree was generated by Tree Dyn program (v198.3) proposed by Methods and Algorithms for Bioinformatics LIRMM. (http://phylogeny.lirmm.fr/phylo_cgi/simple_phylogeny.cgi).

### 3.14. Statistical Analysis

Results are expressed as the mean±standard (SD). Statistical differences were analyzed by one way analysis of variance (ANOVA) using SPSS software (version 13) and p values < 0.05 were considered to be statistically significant.

## 4. Results

### 4.1. Selection of Vaginal Lactobacilli and H_2_O_2_ Production

**Figure 1:**
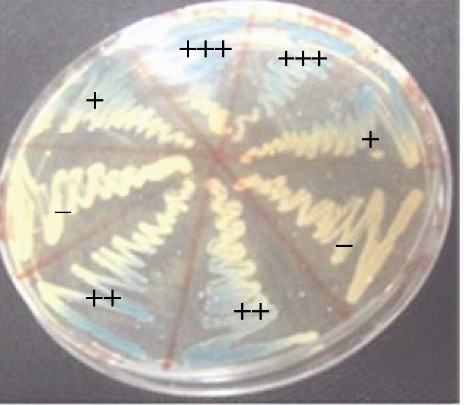
Identification of the hydrogen peroxide production by the isolated lactobacilli: (+++) high production; (++) medium production; and (+) low production. (-) indicates no production

Using Nugent Scoring System, twenty four women (40%) were classified as having normal vaginal flora, 16 (26.66%) were intermediate and 20 (33.33%) were BV. 40 samples that have score <7 were retained. From these samples, 70 lactobacilli isolates were obtained. In this study we used the capacity of producing H_2_O_2_ as the first criterion of selection. As shown in (Figure 1), potential production of H_2_O_2_ capacity was divided into 4 groups according to the intensity of the color. Among the 70 isolates, 57 (81.42%) were found to be able to produce H_2_O_2_ and 26 (37.14%) were hyperproducers.

In this study, isolates producing H_2_O_2_ were selected to evaluate their potential traits.

### 4.2. Survival Under Acidic, Bile and SIF Conditions

Most isolates show considerable tolerance to acid condition with different percentage, where strains B6, B10, and B9 had high tolerance with 82 .23%; 79.98%, and 69.08% survival rates respectively (Table 1).

**Table 1:** Effect of acidic conditions (pH 2.0) bile salts (0.3%) and simulated human intestinal fluid on the survival of vaginal Lactobacillus isolates

**Isolates**	**survival rate (%) ** **pH 2.0**		**survival rate (%) ** **bile salts (0.3%)**		**survival rate (%)** **simulated human intestinal fluid**		
	**0h**	**2h**	**0h**	**8h**	**0h**	**90min**	**180 min**
A1	100	67,71	100	52,91	100	60.43	45.54
A2	100	48,13	100	55,76	100	79.72	58.88
A3	100	54,84	100	71,84	100	73.90	37.41
A4	100	59,72	100	30,96	100	46.51	27.06
A5	100	50,65	100	56,14	100	51.96	31.68
B1	100	72,43	100	35,13	100	52.26	23.56
B3	100	40,22	100	53,36	100	86.34	63.95
B4	100	62,95	100	31,90	100	67.47	51.40
B6	100	82,23	100	77,38	100	74.44	67.41
B9	100	69,08	100	36,52	100	56.60	34.89
B10	100	79,98	100	59,54	100	71.93	60.59
C3	100	62,83	100	51,77	100	62.95	41.68
C5	100	55,70	100	39,97	100	55.43	26.48
W5	100	44,76	100	36.00	100	55.71	43.54

Concerning the resistance of the same isolates to 0.3% bile-salts for 2 h, an important viability was noted. It was 71.84% for strain A3, 77.38% for B6, and 59.54% for B10 (Table 1). The results showed the high differences between strains after incubation for 180 min in SIF. Isolate B10 represent 71.93% survival rate after 90 min of incubation and reached to 60.59% after 180 min (Table 1). It’ is appearing that strains B6 and B10 have the ability to resist to hostiles conditions.

### 4.3. Adhesion to Vaginal Epithelial Cells, Aggregation, Hydrophobicity, and Coaggregation

**Figure 2:**
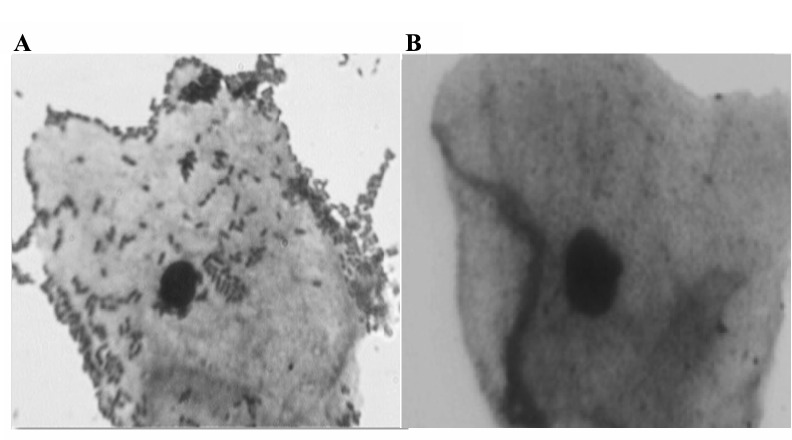
A: Adherence of isolate B6 to vaginal epithelial cells. B: Control

The highest levels of adhesion was observed for the strains B6 (Figure 2), and C3 with the means of 76, and 80 bacteria per VEC, respectively, while other strains such as A1, A3, B9, B10, C5, and W5 adhered less well to VEC with a mean adherence of 60 bacteria at most (Table 2).

**Table 2:** Ability of *Lactobacillus* isolates to adhere to vaginal epithelial cells, percentage of auto aggregation percentage of hydrophobicity and Coaggregation ability.

**Isolates**	**adherents to VECs **	**Autoaggregation (%) (bacteria/ VECs) **					** Hydrophobicity (%) **	**Coaggregation after 5h of incubation (%) **	
		**1h**	**2h**	**3h**	**4h**	**5h**		***S. aureus *** * *(**Vaginal)**	***E. coli *** **(Vaginal)**
A1	65	40.81	45.69	58.05	62.93	67.82	23.33	04.16	03.35
A2	45	25.00	46.88	48.44	50.00	51.03	13.87	15.33	26.02
A3	67	33.14	45.94	46.09	53.62	55.51	17.11	35.17	24.26
A4	33	5.45	9.09	16.36	35.5	45.09	10.70	22.01	12.06
A5	52	10.81	12.74	33.59	41.98	43.85	29.34	14.89	33.87
B1	53	17.59	24.07	31.48	32.41	47.22	8.29	08.25	07.35
B3	45	27.44	35.00	58.31	62.26	63.59	45.15	54.02	04.92
B4	50	15.00	27.00	33.00	52.50	55.00	00.00	14.66	30.65
B6	76	35.00	45.92	47.00	68.00	73.00	34.06	52.12	25.80
B9	68	12.99	49.15	50.05	54.24	58.76	16.32	27.66	37.92
B10	63	10.15	17.00	42.00	57.28	69.50	14.63	35.00	38.05
C3	80	16.32	27.63	43.16	49.47	63.74	00.00	38.88	31.70
C5	68	14.73	14.73	41.09	41.86	48.06	10.20	16.33	06.04
W5	67	27.15	32.65	44.66	50.00	50.22	32.60	33.82	31.17

The results showed that the isolates adhered to VEC with varying degrees, where isolates C3, B6, and B10 were the most adhesives. Bacterial aggregation between microorganisms is one of the important defense mechanism against infection of UGT. Strongly autoaggregating strains A1, A3, B6, B3, B4, B9, B 10, and C3 showed a high autoaggregation percentage between 53 and 73% (Table 2).

The percentages of hydrophobicity toward xylene are shown in (Table 2). Hydrophobic cell surface was demonstrated by high adherence to xylene, an apolar solvent. Our results demonstrated that all tested strains had lower hydrophobicity, where the highest percentage is represented by strain B6 with 34.06%.

In the present study, no correlation was observed between cell surface hydrophobicity, ability to adhere to the vaginal cell and auto aggregation because all strains have low hydrophobicity while some isolates have a very strong ability of adhesion.

The results of co aggregation showed that the highest *S. aureus* coaggregation percentage were obtained with strains B3 (54.02%) and B6 (52.12%) after 5 h of incubation, respectively. The co aggregation with *E. coli* is lower*,* where 38.05% is the highest percentage with strain B10 (Table 2).

### 4.4. Growth Inhibition of Vaginal and Urogenital Pathogens

The results showed that our isolates have exhibited inhibitory activity against pathogenic strains with a significant difference (p<0.05) (Table 3). The smallest diameter of inhibition zone was 9 mm and the widest was 20.5 mm. Most strains present no inhibitory activity against *C. albicans* except strains B6, B1 B4, B10, C3 and C5. However the majority of lactobacilli have antagonistic activity against other pathogens.

**Table 3:** Growth inhibition zones of vaginal, urinary and ATCC strains caused by some lactobacilli and supernatants Strains and supernatants. Mean zone of inhibition of the indicator strains (mm)

**Strains and supernatants**	**Vaginal strains**			**ATCC strains**			**Urinary strains**	
	***E.coli***	***Staphylococcus.*** **sp**	***Candida.*** **sp**	***E.coli*** **ATTC25922**	***S.aureus*** **ATTC25923**	***K.pneumonia*** **ATTC 700603**	***E.coli***	***Klebsiella.*** **sp**
A1	10.5±0.5	14.0 ±1.0	00 ± 00	14.5±0.5	14.5±0.5	12.0 ±1.0	14.5±1.5	13.5±0.5
S.A1	00 ± 00	00 ± 00	00 ± 00	12.5±0.5	00 ± 00	00 ± 00	00 ± 00	12.0 ±1.0
A2	10.0 ±1.0	15.5±0.5	00 ± 00	14.5±1.5	13.5±0.5	10.5±0.5	13.0 ±1.5	11.5±0.5
S.A2	00 ± 00	00 ± 00	00 ± 00	14.0 ±1.0	00 ± 00	08.0 ±1.0	00 ± 00	12.0 ±0.0
A3	11.5±0.5	12.0 ±1.0	00 ± 00	00 ± 00	15.0 ±2.0	12.5±1.5	13.5±2.5	13.5±0.5
S.A3	00 ± 00	00 ± 00	00 ± 00	15.5±0.5	00 ± 00	10.5±0.5	00 ± 00	12.0 ±0.0
A4	00 ± 00	15.5±1.5	00 ± 00	12.0 ±1.0	14±0.0	00 ± 00	11.5±0.5	9.5±1.5
S. A4	00 ± 00	00 ± 00	00 ± 00	00 ± 00	00 ± 00	00 ± 00	00 ± 00	14.5±0.5
A5	12.0 ±1.0	14.5±0.5	00 ± 00	14.5±0.5	13.5±0.5	13.5±0.5	12.5±0.5	13.5±1.5
S.A5	00 ± 00	00 ± 00	00 ± 00	14.5±0.5	00 ± 00	00 ± 00	00 ± 00	12.5±0.5
B1	12.5±0.5	11.0 ±1.0	9.0 ±1.0	12.0 ±2.0	00 ± 00	15.5±0. 5	8.5±0.5	12.0 ±1.0
S.B1	00 ± 00	00 ± 00	00 ± 00	11.0 ±1.0	00 ± 00	12.0 ±1.0	00 ± 00	00 ± 00
B3	00 ± 00	00 ± 00	00 ± 00	11.5±0.5	00 ± 00	00 ± 00	00 ± 00	16.5±0.5
S.B3	00 ± 00	00 ± 00	00 ± 00	00± 00	00 ± 00	00 ± 00	00 ± 00	9.0 ±1.0
B4	08.5±0.5	12.5±0.5	11.5±0. 5	11.0 ±0.0	12.0 ±1.0	00 ± 00	13.5±0.5	9.0 ±1.5
S.B4	00 ± 00	00 ± 00	00 ± 00	12.0 ±1.0	00 ± 00	00 ± 00	13.0 ±1.0	12.0 ±1.0
B6	15.5±1.5	12.0 ±2.0	14.0 ±1.0	00 ± 00	10.5±1.5	13.5±1.5	14.5±0.5	9.5±0.5
S.B6	00 ± 00	14.0 ±1.0	10.0 ±1.0	00 ± 00	11.0 ±1.0	00 ± 00	00 ± 00	13.5±0.5
B9	00 ± 00	8.0 ±3.0	00 ± 00	15.0 ±2.0	00 ± 00	00 ± 00	00 ± 00	13.5±0.5
S.B9	00 ± 00	00 ± 00	00 ± 00	00 ± 00	00 ± 00	00 ± 00	00 ± 00	12.5±2.5
B10	15.0 ±1.0	16.5±0.5	10.5±1.0	10.5±1.5	14.5±0.5	11±1	14.5±0.5	12.5±0.5
S. B10	00 ± 00	12.0 ± 0.1	9.0 ± 0.1	00 ± 00	00 ± 00	00 ± 00	13.0 ±2.0	13.0 ±0.0
C3	12.0 ±1.0	12.0 ±1.0	14.0 ±1.0	14.0 ±1.0	13.0 ±1.0	00 ± 00	12.5±0.5	00 ± 00
S.C3	00 ± 00	00 ± 00	00 ± 00	12.5±0.5	00 ± 00	00 ± 00	12.5±0.5	00 ± 00
C5	14.5±1.5	16.0 ±1.0	11.5±1.5	15.5±0.5	13.0 ±1.0	00 ± 00	13.0 ±1.0	11.0 ±1.0
S.C5	00 ± 00	00 ± 00	00 ± 00	15.5±0.5	00 ± 00	00 ± 00	00 ± 00	00 ± 00
W5	13.0 ± 1.0	20.5±1.5	00 ± 00	16.5±0.5	23.5±0.5	13.5±0.5	15.5±0.5	12.5±0.5
S.W5	10.0 ± 1.0	00 ± 00	00 ± 00	13.5±0.5	00 ± 00	11.0 ± 0.0	11.5±0.5	13.5±0.5

S: Supernatant

The results showed that *Klebsiella.*sp from urinary origin was inhibited by all cultures and their supernatants except supernatants of B1, C3, C5, and the culture of strain C3. Inhibition zones were shown to be produced by lactobacilli, as they disappeared when we used the supernatant of the most isolates with the exception for supernatant obtained from a number of strains such as B6 and B10 which have the ability of inhibiting vaginal and urinary pathogens.

### 4.5. Antibiotic Resistance

The results showed that all strains were resistant to Ofloxacin, Gentamicin, and Ciprofloxacin; with almost all strains were sensitive to Trimethoprim- Sulphamethoxazole, Ampicillin, Erythromycin, Cefotaxime, Chloramphenicol, Tetracycline and Nitrofurantoin (Table 4).

**Table 4:** Antibiotic susceptibility of some vaginal lactobacilli

** Isolates**	** Antibiotics**									
	**SXT**	**AM**	**OFX**	**GEN**	**CIP**	**ERY**	**CTX**	**CHL**	**TET**	**NIT**
	**Inhibition zone (mm)**									
A1	25.5±0.5 ^S^	22.5±0.5 ^S^	00 ^R^	09.5±1.5 ^R^	00 ^R^	25.5±0.5 ^S^	30.0±2.0 ^S^	33.0±1.0 ^S^	21.0±0.0 ^S^	31.5±1.5 ^S^
A2	22.5±2.5 ^S^	23.0 ±1.0 ^S^	00 ^R^	10.5±0.5 ^R^	00 ^R^	25.0 ±1.0 ^S^	27.5±2.5 ^S^	33.0±0.0 ^S^	17.5±1.5 ^I^	32.5±1.5 ^S^
A3	26.0±1.0 ^S^	23.0 ±2.0 ^S^	00 ^R^	09.0 ±1.0 ^R^	00 ^R^	23.5±0.5 ^S^	27.0±0.0 ^S^	31.0±1.0 ^S^	21.0±1.0 ^S^	30.0±1.0 ^S^
A4	23.5±0.5 ^S^	23.0 ±4.0 ^S^	00 ^R^	10.5±1.0 ^R^	00 ^R^	24.5±1.5 ^S^	27.5 ±2.5^S^	33.5±0.5 ^S^	22.0±0.0 ^S^	32.5±0.5 ^S^
A5	24.5±2.5 ^S^	25.0 ±1.0 ^S^	00 ^R^	10.0 ±0.0 ^R^	00 ^R^	23.5±1.5 ^S^	25.0±2.0 ^S^	29.0±1.0 ^S^	20.5±0.5^I^	29.5±0.5 ^S^
B1	25.0±1.0 ^S^	21.5±1.5 ^S^	00 ^R^	10.0 ±0.0 ^R^	00 ^R^	22.5±2.5^S^	23.0 ±2.0 ^S^	29.0±0.0 ^S^	20.5±0.5^I^	31.0±0.0 ^S^
B3	24.5±1.5 ^S^	24.5±1.5 ^S^	00 ^R^	09.5±0.5 ^R^	00 ^R^	21.5±1.5 ^S^	25.0±2.0 ^S^	27.5±2.5 ^S^	20.0±0.0 ^I^	29.0±1.0 ^S^
B4	25.5±0.5 ^S^	21.5±1.5 ^S^	00 ^R^	11.5±0.5 ^R^	00 ^R^	21.0 ±1.0 ^S^	20.0±3.0^I^	29.5±0.5 ^S^	18.0 ±2.0 ^I^	29.0±0.0 ^S^
B6	26.0±1.0 ^S^	24.5±0.5 ^S^	00 ^R^	10.5±0.5 ^R^	00 ^R^	25.0 ±0.0 ^S^	26.5±0.5 ^S^	31.0±1.0 ^S^	20.5±0.5 ^I^	31.0±0.0 ^S^
B9	23.5±0.5^S^	21.5±1.5 ^S^	00 ^R^	12.0 ±0.0 ^R^	00 ^R^	25.0 ±0.0 ^S^	25.5±0.5 ^S^	30.5±0.5 ^S^	20.5±0.5 ^I^	30.0±0.0 ^S^
B10	25.5±1.5 ^S^	24.5±0.5 ^S^	00 ^R^	11.5±0.5 ^R^	00 ^R^	24.5±0.5 ^S^	29.0±1.0 ^S^	32.5±1.5 ^S^	23.5±0.5 ^S^	32.5±0.5 ^S^
C3	23.0±2.0^S^	15.0±0.5 ^R^	00 ^R^	10.5±0.5 ^R^	00 ^R^	25.0 ±0.0 ^S^	28.5±1.5 ^S^	30.5±0.5 ^S^	21.0±0.0 ^S^	32.5±2.5 ^S^
C5	25.0±1.0 ^S^	20.0 ±1.0 ^I^	00 ^R^	10.5±0.5 ^R^	00 ^R^	23.5±1.5 ^S^	28.5±3.5 ^S^	32.5±2.5 ^S^	20.0±0.0 ^I^	31.0±1.0 ^S^
W5	24.5±0.5 ^S^	25.0 ±0.0 ^S^	00 ^R^	10.5±0.5 ^R^	00 ^R^	21.0 ±1.0 ^S^	27.0±2.0 ^S^	33.0±1.0 ^S^	21.0±1.0 ^S^	32.0±1.0 ^S^

(STX) Trimethoprim-sulphamethoxazole , (AM) Ampicilin , (OFX) Ofloxacin , (GEN) Gentamicin , (CIP) Ciprofloxacin , (ERY) Erythromycin , (CTX) Cefotaxime, (CHL) Chloramphenicol , (TET) Tetracycline , (NIT) Nitrofurantoin.

### 4.6. Resistance of Vaginal Lactobacilli to Spermicides and Acid Production

The results of the test are summarized in Table 5. We found that all strains show not an inhibition zone with the first two concentrations (0.1%, 0.2%), while when a concentration increases to 1%, inhibition zones between 3.5 and 7.5 mm could be measured for 12 strains, except B10 and B6 isolates. A significant inhibition was noted with 2.5% where zones are in majority > 6.5 mm. Through these results, we conclude that most of the strains are resistant to the tested spermicide and are able to be co-administered with this spermicide for the treatmentof vaginal infections.

**Table 5:** Resistance of vaginal strains to spermicides and acid production

**Isolates**	**Concentration of spermicides (%)**				**Acid production ** **Control broth ( pH 5.85)**		
	**0.1**	**0.25**	**1**	**2.5**	**4h**	**6h**	**24h**
A1	00	00	7.0±0.0	7.5±0.5	5.45±0.010	5.19±0.050	4.07±0.010
A2	00	00	3.5± 3.5	7.0±1.0	5.55±0.005	5.25±0.010	4.09±0.010
A3	00	00	7.0±0.0	8.0±0.0	5.56±0.010	5.23 ±0.020	4.08±0.010
A4	00	00	8.0±0.0	11.5±1.5	5.59±0.005	4.95±0.010	4.04±0.010
A5	00	00	6.5±0.5	9.5±1.5	5.57±0.010	4.93±0.020	4.03±0.010
B1	00	00	3.5±3.5	9.0±2.0	5.67±0.020	4.97±0.010	4.04±0.005
B3	00	00	6.5±0.5	7.0±1.0	5.64±0.010	4.90±0.010	4.05±0.020
B4	00	00	7.5±0.5	7.5±0.5	5.63±0.005	4.90 ±0.030	4.03±0.005
B6	00	00	00± 0.0	7.5±0.5	5.64±0.010	4.99±0.010	4.05 ±0.005
B9	00	00	5.5±0.5	7.5±0.5	5.59±0.005	4.94±0.020	4.02±0.005
B10	00	00	00± 0.0	7.0± 0.0	5.41±0.005	5.14±0.005	4.17±0.030
C3	00	00	6.5±1.5	6.5±1.5	5.49±0.020	5.25±0.005	4.10±0.005
C5	00	00	7.5±0.5	7.5±0.5	5.57±0.010	5.30±0.010	4.13 ±0.005
W5	00	00	6.5±0.5	9.5±1.5	5.57 ±0.020	5.32±0.030	4.11 ±0.005

The results showed that all lactobacilli have an acidifying capacity with a significant difference between strains (p <0.05). Measuring the initial pH, and the pHs after 4 h, 6 h, and 24 h of incubation shows a decrease in the pH of the each sample and development of an acid environment with a pH∼ 4.0 that contributes to the maintenance of the high redox potential which can protect the vagina against the invasion of the undesirable microorganisms (Table 5).

### 4.7. Identification of the Selected Isolates

**Figure 3:**
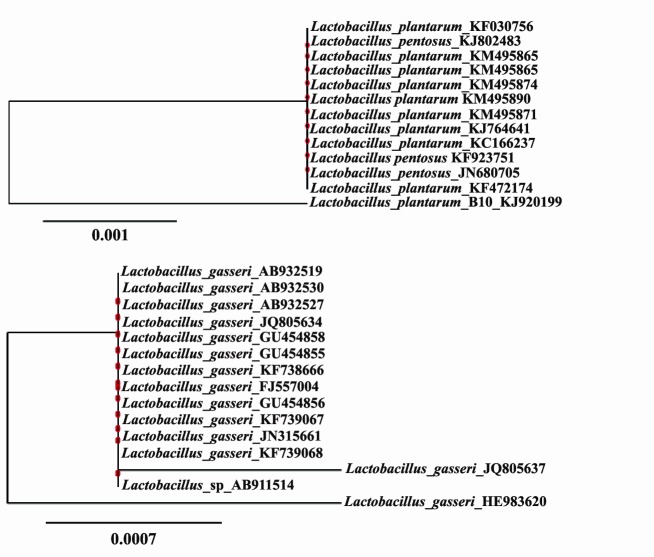
Clustering of *Lb. plantarum* B10 and *Lb. gasseri* B6

Two strains were retained as they display a high potential of probiotic profile, they were identified by 16S rDNA gene sequences and showed a high similarity to *Lb. gasseri* (B6: 99%) and to *Lb. plantarum* (B10: 99%). The GenBank accession numbers of the isolates B6 and B10 are assigned as KF739067 and KJ920199, respectively (Figure 3).

## 5. Discussion

In our study, 14 isolates producing H_2_O_2_ were chosen to evaluate their technological and probiotic properties. The production of H_2_O_2_ by lactobacilli is considered as a non specific antimicrobial defense mechanism of the normal vaginal ecosystem (20) since women colonized by H_2_O_2_ producing strains had a protective effect against bacterial vaginosis (28-30). Aroutcheva *et al* (17) reported that 81.80% of the isolated strains produce H_2_O_2_ where 38.90% had a high H_2_O_2_ production. Several studies have demonstrated that *Lactobacillus* species can be administered orally or vaginally resulting in colonization in vagina, reduction in vaginal coliform counts and even reduction in UGTI (31). Therefore, we studied their viability under acidic, bile and SIF conditions since these abilities are important if the strains are recommended to be used orally as therapeutic probiotic. To exert their beneficial effect, lactobacilli need to resist human gastric transit conditions (22,32). Our results showed that strains B6 and B10 have the highest tolerance to the gastro-intestinal condition. The results of a similar study have also shown the tolerance of vaginal *Lb. fermentum* SK5 to stimulated human gastrointestinal tract conditions (22).

The ability to adhere to the epithelial cells and coaggregation ability are considered as important criterions for *in vitro* probiotic selection (15,33,34). Our results are in agreement with those of several authors (10,14). Similarly, Strus *et al* (10) showed that from 111 isolates, 50% have a high adherence to vaginal mucus.

The relationship between autoaggregation, adhesion and hydrophobicity has been reported by several authors (7,22,25,35), however these correlations have not been found or reported by the others (36,37). The obtained results were in agreement with those reported by Blakrishma (36); as he showed that there is no correlation between these parameters. In another study, Lp9 isolate has surface hydrophobicity of 37-38%, suggesting its adhesiveness (37). Furthermore, adhesion and coaggregation of lactobacilli probiotic may inhibit the adherence of pathogens to the tissue receptors on the vaginal epithelial cells (12,38).

Our isolates present antagonistic activity against indicator bacteria. H_2_O_2_, lactic acid and other organic acids produced by LAB are frequently associated with this activity *in vitro* (15). The inhibition of urogenital infections increases the relevance of these wild strains for use in probiotic products (39). In another study different inhibitory ability between vaginal lactobacilli strains against *C. albicans* were obtained (40).

It is very important to know the susceptibility of vaginal LAB to antibiotics in order to understand their behavior with respect to antibiotics in pharmaceutical preparations which were used for restoration of unbalanced vaginal flora (16). Probiotic strains’ resistance antibiotic may be an advantage in the case of coadministrations, but not all lactobacilli have intrinsic resistance (22).

According to our results, the majority of strains are resistant to the tested spermicide. Pascual *et al*. (20) have studied the resistance and susceptibility of 62 strains of LAB to spermicide and have shown that 19.4% were resistant, whereas 80.6% were sensitive to nonoxynol-9*.*

The result showed that *Lb. gasseri* B6 produces a high quantity of the lactic acid. It was reported that production of organic acid by LAB in the vaginal environment is responsible for the change in the pH and when the vaginal pH was lower than 4.5 it reflects a healthy vaginal ecosystem (9,17,41). According to Aslim and Kilic (19) lactobacilli produce lactic acid to maintain vaginal pH≤4.5; this mechanism is used against pathogenic microorganisms.

## 5. Conclusions

In conclusion, *Lb. gasseri* B6 and *Lb. plantarum* B10 possessed desirable technological and probiotic properties. These *Lactobacillus* isolates are the best candidate for preventing vaginal and urogenital infections.

## Acknowledgments

This work was supported by a grant allocated by the “Ministère de l’Enseignement Supérieur et de la Recherche Scientifique” of Algeria (Project code: F01720120001).

## References

[1] Atassi F, Brassar D, Grob Ph, Servin AL (2006a). Lactobacillus strains
isolated from the vaginal microbiota of healthy women inhibit
Prevotellabivia and Gardnerella vaginalis in coculture and
cell culture. FEMS Immunol Med Microbiol.

[2] Atassi F, Brassar D, Grob Ph, Graf F, Servin AL (2006b). Vaginal Lactobacillus isolates inhibit uropathogenic Escherichia coli. FEMS Microbiol Lett.

[3] Zárate G, Nader-Macias M (2006). Influence of probiotic vaginal lactobacilli on in vitro adhesion of urogenital pathogens to vaginal epithelial cells. Lett Appl Microbiol.

[4] Gardiner G, Heinemann C, Bruce A, Beuerman D, Reid G (2002). Persistence of Lactobacillus fermentum RC-14 and L rhamnosus GR-1 but not L rhamnosus GG in the human vagina as demonstrated by randomly amplified polymorphic DNA. Clin Diagn Lab Immunol.

[5] Voravuthikunchai SP, Bilasoi S, Supamala O (2006). Antagonistic activity against pathogenic bacteria by human vaginal lactobacilli. Anaerobe.

[6] Garg k B, Ganguli I, Das R, Talwar GP (2009). Spectrum of Lactobacillus species present in healthy vagina of Indian Women. Indian J Med Res.

[7] Kaewsrichan J, Peeyananjarassri K, Kongprasertkit J (2006). Selection and identification of anaerobic lactobacilli producing inhibitory compounds against vaginal pathogens. FEMS Immunol Med Microbiol.

[8] Gil NF, Martinez RCR, GomesCB GomesCB, NomizoA NomizoA, De Martinis ECP (2010). Vaginal lactobacilli as potential probiotic against Candida ssp. Braz J Microbiol.

[9] Dimitonova SP, Danova ST, Serkedjieva JP, Bakalov BV (2007). Antimicrobial activity and protective properties of vaginal lactobacilli from healthy Bulgarian women. Anaerobe.

[10] Strus M, Kucharska A, Kukla G, Brzychczy MW, Maresz K, Heczkoi PB (2005). The in vitro activity of vaginal Lactobacillus with probiotic properties against Candida. Infect Dis Obstet Gynecol.

[11] EO’Hanlon D, Moench TR, Cone RA (2011). In vaginal fluid, bacteria associated with bacterial vaginosis can be suppressed with lactic acid but not hydrogen peroxide. BMC Infect Dis.

[12] BorisS BorisS, Suarez JE, Vazquez F, Barbés C (1998). Adherence of Human Vaginal Lactobacilli to Vaginal Epithelial Cells and Interaction with Uropathogens. Infect Immunol.

[13] Ocaña VS, Bru E, Ruiz A, Holgado AP, Nader-Macias ME (1999). Surface characteristics of lactobacilli isolated from human vagina. J Gen Appl Microbiol.

[14] Osset J, Bartolome RM, Garcia E, Andreu A (2001). Assessment of the capacity of Lactobacillus to inhibit the growth of uropathogens and block their adhesion to vaginal epithelial cells. J Infect Dis.

[15] Fraga M, Perelmuter K, Delucchi L, Cidade E, Zunino P (2008). Vaginal lactic acid bacteria in the mare: evaluation of the probiotic potential of native Lactobacillus spp and Enterococcus spp Strains. Antonie Van Leeuwenhoek.

[16] Ayenalem S, Yusuf L, Ashenafi M (2010). Lactic Acid Bacterial Vaginosis among Outpatients in Addis Ababa. Ethiop J Health Dev.

[17] Aroutcheva A, Gariti D, Simon M, Shott S, Faro J, Simoes JA (2001). Defense factors of vaginal lactobacilli. Am J Obstet Gynecol.

[18] Reid G (2000). In vitro testing of Lactobacillus acidophilus NCFM as a possible probiotic for the urogenital tract. Int Dairy J.

[19] Aslim B, Kilic E (2006). Some probiotic properties of vaginal lactobacilli isolated from healthy women. Jpn J Infect Dis.

[20] Ascual LM, Daniele MB, Pajaro C, Barberis L (2006). Lactobacillus species isolated from the vagina: identification, hydrogen peroxide production and nonoxynol-9 resistance. Contraception.

[21] Yixu H, Tian W, Wan C, Jia L, Wang L, Yuan J (2008). Antagonistic Potential against Pathogenic Microorganisms and Hydrogen Peroxide Production of Indigenous Lactobacilli Isolated from Vagina of Chinese Pregnant Women. Biomed Environ Sci.

[22] Kaewnopparat S, Dangmanee N, Kaewnopparat N, Srichana T, Chulasiri M, Settharaksa S (2013). In vitro probiotic properties of Lactobacillus fermentum SK5 isolated from vagina of a healthy woman. Anaerobe.

[23] Woraharn S, Chaiyasut C, Sirithunyalug B, Sirithunyalug J (2010). Survival enhancement of probiotic Lactobacillus plantarum CMU-FP002 by granulation and encapsulation techniques. Afr J Microbiol Res.

[24] Mclean NW, Rosenstenin IJ (2000). Characterization and selection of Lactobacillus species to re-colonise the vagina of women with recurrent bacterial vaginosis. J Med Microbiol.

[25] Kos B, Šuškovic J, Vukoviæ S, Šimpraga M, Frece J, Matošiæ S (2003). Adhesion and aggregation ability of probiotic strain Lactobacillus acidophilus M92. J Appl Microbiol.

[26] Iyer R, Tomar SK, Kapila S, Mani J, Singh R (2010). Probiotic properties of folate producing Streptococcus thermophilus strains. Food Res Int.

[27] Vlkova´ E, Rada V, Popela´r¡ova´ P, Trojanova´ I, Killer J (2006). Antimicrobial susceptibility of bifidobacteria isolated from gastrointestinal tract of calves. Livest Sci.

[28] Dasari S, Shouri RND, Wudayagiri R, Valluru L (2014). Antimicrobial activity of Lactobacillus against microbial flora of cervico vaginal infections. Asian Pac J Trop Dis.

[29] Vallor AC, Antonio MAD, Hawes SE, Hillier SL (2001). Factors associated with acquisition of, or persistent colonization by,vaginal lactobacilli: role of hydrogen peroxide production. J Infect Dis.

[30] Merk K, Borelli C, Korting HC (2005). Lactobacilli-bacteria-host interactions with special regard to the urogenital tract. Int J Med Microbiol.

[31] Reid G, Burton J, Devillard E (2004). The Rationale for Probiotics in Female Urogenital Healthcare. Med Gen Med.

[32] Vinderola CG, Reinheimer JA (2003). Lactic acid starter and probiotic bacteria: a comparative in vitro study of probiotic characteristics and biological barrier resistance. Food Res Int.

[33] Nivoliez A, Camares O, Paquet- Gachinat M, Bornes S, Forestier Ch, Veisseire PH (2012). Influence of manufacturing processes on in vitro properties of the probiotic strain Lactobacillus rhamnosus Lcr35. J Biotechnol.

[34] Ekmekci H, Aslim B, Ozturk S (2009). Characterization of vaginal lactobacilli coaggregation abbility with Escherichia coli. Microbiol Immunol.

[35] Collado MC, Meriluoto J (2008). Adhesion and aggregation properties of probiotic and pathogen strains. Eur Food Res Technol.

[36] Balakrishna A (2013). In vitro evaluation of adhesion and aggregation abilities of four potential probiotic strains isolated from guppy (Poeciliareticulata). Braz Arch Biol Technol.

[37] Kaushik JK, Kumar A, Duary RK, Mohanty AK, Grover S, Batish VK (2009). Functional and Probiotic Attributes of an Indigenous Isolate of Lactobacillus plantarum. PLoSONE.

[38] Mastromarino P, Brigidi P, Macchia S, Maggi L, Pirovano F, Trinchieri V (2002). Characterization and selection of vaginal Lactobacillus strains for the preparation of vaginal tablets. J Appl Microbiol.

[39] Juàrez Tomàs MS, Ocana SV, Wiese B, Nader-Macias ME (2003). Growth and lactic acid production by vaginal Lactobacillus acidophilus CRL 1259 and inhibition of uropathogenic Escherichia coli. J Med Microbiol.

[40] Rousseau V, Lepargneurb JP, Roquesc C, Remaud-Simeond M, Paul F (2005). Prebiotic effects of oligosaccharides on selected vaginal lactobacilli and pathogenic microorganisms. Anaerobe.

[41] Matu MN, Orinda GO, Njagi ENM, Cohen CR, Bukusi EA (2010). In
vitro inhibitory activity of human vaginal lactobacilli against pathogenic
bacteria associated with bacterial vaginosis in Kenyan
women. Anaerobe.

